# Multi-Objective Optimization of Energy Saving and Throughput in Heterogeneous Networks Using Deep Reinforcement Learning

**DOI:** 10.3390/s21237925

**Published:** 2021-11-27

**Authors:** Kyungho Ryu, Wooseong Kim

**Affiliations:** Department of Computer Engineering, Gachon University, Seongnam 13120, Korea; rudgh1368@gachon.ac.kr

**Keywords:** wireless heterogeneous network, energy saving, wireless backhaul mesh, deep reinforcement learning

## Abstract

Wireless networking using GHz or THz spectra has encouraged mobile service providers to deploy small cells to improve link quality and cell capacity using mmWave backhaul links. As green networking for less CO_2_ emission is mandatory to confront global climate change, we need energy efficient network management for such denser small-cell heterogeneous networks (HetNets) that already suffer from observable power consumption. We establish a dual-objective optimization model that minimizes energy consumption by switching off unused small cells while maximizing user throughput, which is a mixed integer linear problem (MILP). Recently, the deep reinforcement learning (DRL) algorithm has been applied to many NP-hard problems of the wireless networking field, such as radio resource allocation, association and power saving, which can induce a near-optimal solution with fast inference time as an online solution. In this paper, we investigate the feasibility of the DRL algorithm for a dual-objective problem, energy efficient routing and throughput maximization, which has not been explored before. We propose a proximal policy (PPO)-based multi-objective algorithm using the actor-critic model that is realized as an optimistic linear support framework in which the PPO algorithm searches for feasible solutions iteratively. Experimental results show that our algorithm can achieve throughput and energy savings comparable to the CPLEX.

## 1. Introduction

Exponentially increasing mobile traffic accelerates the deployment of dense small cells operating on the 3 GHz spectrum under legacy macro cells, called a heterogeneous small cell network (HetNet), which offloads congested macro cells and eventually enhances quality of user experience (QoE). User equipments (UEs) can have dual connectivity to the macro eNB (MeNB) and small eNB (SeNB) for control/data bearer splitting or download busting. Such SeNB deployment is costly when backhauling to a network gateway (a MeNB in this paper). Millimeter-wave (mmWave)-based backhauling can reduce deployment efforts and provide gigabit data rates to UEs using huge bandwidths, such as 9 and 10 GHz, available at the 60 GHz band and E-band. Many measurement campaigns and demonstrations at 28, 38, 60 and 73 GHz have already shown the feasibility of mmWave use for mobile communication [1,2,3].

**Figure 1:**
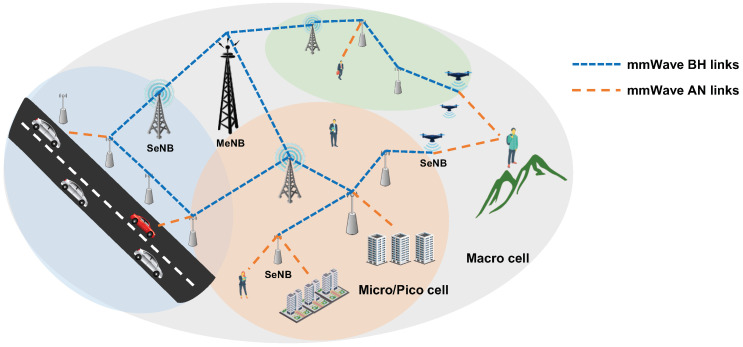
Heterogeneous cellular network architecture with mmWave backhaul mesh.

To overcome the short communication range of the mmWave link due to its high pathloss and low penetration, beam forming based on directional antennae and repeaters for amplifying is necessarily considered. Figure 1 shows the HetNet equipped by a multi-hop backhaul mesh network for long-range backhauling of the mmWave links, in which an SeNB unreachable by the MeNB can access the Internet through multi-hop relays of the SeNBs [4,5]. The mmWave-based backhaul mesh networks have several challenges, such as efficient radio resource management (RRM) [5,6], interference management [7,8], multi-hop routing [9], and energy saving [10].

Due to increasing power consumption from excessively deployed SeNBs and mmWave backhaul transmissions, various approaches to save energy in mobile networks have been considered [11]; these include switching off small and macro cells [12,13,14] or adjusting cell size dynamically [15,16], where users of switched-off SeNBs are supported by neighboring SeNBs using remaining resources.

Especially for the HetNets with mmWave-based backhauls, Chen et al. [17] introduced a user association and power allocation algorithm for energy harvesting and self-backhaul SeNB to maximize energy efficiency. Additionally, Mesodiakaki et al. [18] studied an energy- and spectrum-efficient user association problem considering mmWave backhauls. Hao et al. [19] investigated the energy-efficient resource allocation in two-tier massive multiple-input multiple-output (mMIMO) HetNets with wireless backhauls.

Most previous works focus on radio resource allocation to increase spectral and energy efficiency in the HetNets. However, in the mmWave backhaul mesh, a multi-hop routing mechanism determines energy saving, as SeNBs need to be switched on for relaying regardless of the presence of associated users. We establish a fluid model of user traffic in the mmWave-based backhaul mesh and solve the joint optimization problem that minimizes energy consumption while guaranteeing the demanded data rate of each UE [9]. This problem can be formulated in a non-convex mixed integer linear problem (MILP), known as a NP-hard. When we used the branch-and-cut algorithm of CPLEX to find an optimum in a given HetNet topology, it consumed more than 30 min of calculation time, which is infeasible, as the HetNet topology changed dynamically due to UE mobility. For the online algorithm, previous works [17,18,19] considered heuristic or iterative algorithms, which cannot be guaranteed to find a near optimal solution or can suffer from convergence delays. In this study, we consider a deep reinforcement learning (DRL) algorithm to find a feasible solution of the MILP problem in real time.

Reinforcement learning (RL) [20] has received much attention for dynamic systems, which can provide a long-term solution considering future rewards. Furthermore, the deep learning technique has recently been applied to overcome the curse of dimensionality as the size of the Markov decision process (MDP) increases in terms of state and action space [21,22,23,24,25]. RL based on a deep neural network (DNN) can provide a feasible online solution; feed-forward computation is simple for inference compared to backward computation for training. Thus, many researchers now consider the DRL algorithm to solve NP-hard problems of the wireless communication and networking field.

Recently, many studies about applying DRL to wireless communication problems have been introduced, as in the related work section. Several works [26,27,28,29,30,31,32,33,34,35,36,37] used DRL to allocate radio resources, transmission power and channels to increase spectral efficiency; additionally, multiple access schemes were also exploited by DRL in [38,39,40,41]. For energy saving, several studies developed a DRL algorithm for an energy-efficient multi-hop routing protocol or peer-to-peer connectivity in the ad hoc networks of satellites or UAVs [42,43], where individual mobile agents learn an optimal policy to maintain connectivity while saving limited power. Refs. [44,45,46] introduced energy-saving mechanisms using DRL, wherein an agent controls the transmission power, association and sleep mode of SeNBs in a HetNet without multi-hop backhauls. To the best of our knowledge, this is the first work that investigates DRL to find the Pareto front of a multi-objective optimization problem of energy saving and throughput maximization in the HetNet with an mmWave-based multi-hop backhaul mesh.

Key motivations of this study are enumerated as below:

- There has not been notable research on an energy efficient multi-hop routing algorithm using DRL for an mmWave backhaul mesh of a dense HetNet;
- The DRL-based algorithm can be considered to find a Pareto front solution for the dual-objective optimization of energy saving and throughput maximization in the HetNet.

To solve our optimization problem, we adopt a proximal policy optimization (PPO)-based DRL algorithm [24] which shows typically fast and reliable convergence in the training phase as one of popular policy-based DRL algorithms. The PPO algorithm can provide an online policy for controlling backhaul transmission and SeNB power in HetNets, and it is simple to implement but comparable with the complicated trust region policy optimization (TRPO) [23] in terms of performance. However, it is a challenge for the PPO algorithm to find an optimum of the multi-objective problem if only the reward sum of conflicting multi-objectives is given to an agent for training. Therefore, we consider a multi-objective reinforcement learning (MORL) approach [47] to find the Pareto front solutions.

Optimistic linear support (OLS) is proposed for the MORL [48], in which an outer loop iteratively calls a single-objective solver based on the deep Q-network as a subroutine. In this paper, we propose PPO-based deep optimistic linear support (PDOLS), where the PPO algorithm iteratively solves the scalarized objective problem by a specific weight vector for rewards. In experiments, the proposed PDOLS searched optimal corner weights for multi-objectives efficiently and resulted in similar outcomes to the optimal weights obtained through repeated experiments. Additionally, the PDOLS achieved notable throughput and energy saving compared to the CPLEX results [9]; the CPLEX achieves a 35% energy savings and a 14 Mbps data rate without blockage, while the PDOLS achieves an almost 28% energy savings and a 13.4 Mbps data rate. Such performance reduction is small, considering the CPLEX execution time and DRL inference time are 30 min vs. 1 s. Furthermore, we improve the PDOLS with a scaled reward (PDOLS-SR) that adjusts the reward values according to the environment, which increases the probability of finding the optimal weight vector.

We highlight our key contributions of this study as below:

- We propose a PPO-based online algorithm for the bi-objective problem of energy minimization and throughput maximization;
- We propose an integrated framework based on the PPO algorithm and OLS to find the Pareto front of the two objectives;
- We demonstrate the feasibility of the proposed online solution based on DRL in a HetNet environment.

The remainder of the paper is organized as follows. We introduce recent works on DRL for wireless networking solutions in Section 2, and offer an overview of the DRL background in Section 3. In Section 4, we establish the multi-objective optimization model for energy saving and throughput maximization in HetNets. We propose the PPO and PDOLS algorithm for the multi-objective optimization problem in Section 5. Section 6 shows our experimental results regarding performance of the learning algorithm and HetNet throughput. Finally, we discuss and conclude our study in Section 7.

## 2. Related Works

Previously, most of the NP problems in the wireless communication and networking area were solved by linear approximation or heuristic algorithms, such as simulated annealing (SA), generic algorithm (GA), particle swarm optimization (PSO), etc. Recent successes of the DNN technique in computer vision and speech recognition show the possibility of applying large-scale feed-forward neural networks to wireless networking. Therefore, the 1D or 2D convolution neural network (CNN) that is popular for computer vision and image processing was used for wireless channel estimation with MIMO [49,50,51], automatic modulation and coding schemes [52,53,54] and network intrusion detection [55,56,57,58].

In contrast to the above supervised deep learning, artificial intelligence for controlling dynamics of the wireless networking system needs to be made naturally by past experience in the system. Such dynamic systems can be modelled by the MDP; at each step, a network agent acts based on the state and receives reward feedback for the action, such as successful transmission, packet loss, collision, saving power, etc. Using the collected experience data, the DRL algorithm can effectively find an optimal solution of the wireless networking system. The following studies have demonstrated feasibility of using DRL algorithms for wireless communication and networking during the last several years (refer to the summary in Table 1).

Wang et al. [26] proposed a dynamic multi-channel access mechanism based on deep Q-learning. A node selects one multi-channel that has low interference, which returns the maximum reward for the action. Zhong et al. [27,28] used the actor-critic algorithm to explore the sensing policy for dynamic channel access and considered a multi-agent model for distributed sensors in a partially observable environment. Naparstek et al. [29,30] also proposed DQN-based multi-agents which act based on Q-value independently. Li et al. [31] applied the DQN for channel sensing, and Liu et al. [32] proposed a hierarchical deep Q-network (h-DQN) model for cooperative channel sensing, which divides the original problem into separate sub-problems for multi-DRL agents.

Ali et al. [38] introduced a Q-learning-based MAC protocol in dense WLANs which learns the optimal policy based on channel state and transmission action experience. Yu et al. [39] investigated a DRL-based MAC protocol for heterogeneous wireless networking which was called deep-reinforcement learning multiple access (DLMA). They established a new multi-dimensional RL framework based on the Q-learning that maximizes sum throughput and provides proportional fairness, even co-existing with TDMA-like ALOHA protocols. Al et al. [40] studied radio resource scheduling (RRS) in the cellular MAC layer using the DQN. Nisioti et al. [41] presented a MAC solution for sensor networks based on coordinated reinforcement learning by considering the dependencies among sensors to find the optimal actions.

Zhao et al. [59] studied user association and radio resource allocation in a HetNet. For a large action space, they considered a multi-agent RL approach and a dueling double deep Q-network (D3QN) to obtain an optimal policy with little computation complexity. Zhang et al. [60] proposed a DRL algorithm for the association between each IoT device and a cellular user to maximize the sum rate of all the IoT devices in symbiotic radio networks (SRNs). Ding et al. [61] introduced the user association and power control scheme using the multi-agent DQN to ensure the UE’s quality of service (QoS) requirements.

He et al. [33] proposed an orchestration framework in vehicular networks with a novel DRL algorithm for the resource allocation of networking, caching and computing resources. Shi et al. [34] modelled a hierarchical DRL-based multi-DC (drone cell) trajectory planning and resource allocation scheme for high-mobility users. In [35,36], the authors also conducted resource allocation for uplink nonorthogonal multiple access (NOMA) systems using a DRL-based algorithm to solve the nonconvex optimization problem. Rahimi et al. [37] also tried to increase scalability with a hierarchical DRL for joint user association and resource allocation in the NOMA system.

Liu et al. [43] introduced a novel DRL-based energy-efficient routing protocol called DRL-ER, which avoids the battery energy imbalance of constellations and guarantees a required end-to-end delay bound. Liu et al. [42] adopted a DRL-based energy-efficient control for coverage and connectivity in UAV communication systems. Du et al. [62] reviewed and analyzed how to achieve green DRL for radio resource management (RRM). Dai et al. [63] utilized DRL to design an optimal computation offloading and resource allocation strategy for minimizing energy consumption. El et al. [44] solved the energy-delay-trade-off (EDT) problem in a HetNet where small cells can switch to different sleep mode levels to save energy while maintaining QoS using the DRL.

To the best of our knowledge, our study is first to develop a PPO-based multi-objective algorithm that controls multi-hop routing and switching on/off SeNBs in HetNets, even though many previous works have applied the DRL algorithm for other optimization problems.

## 3. Deep Reinforcement Learning (DRL)

This section provides a brief overview of reinforcement learning (RL) and DRL. RL is a popular machine learning algorithm which allows agents to learn optimal behavior through trial-and-error interactions with a dynamic environment. A key strategy of the RL is utilizing statistics to obtain an optimal control decision (policy) in the form of the MDP. The MDP is modelled by $\left(S,A,P_{ss}^{a},R^{a}\right)$, wherein the state space is represented by *S*, the action space is represented by *A*, the state transition probability is $P_{ss^{\prime }}$ at a taken action *a* and a corresponding reward *R*, and in which the policy as a function π(s) specifies an action *a* in each state *s*. Therefore, an optimal policy, $\pi^{*}$, maximizes the expected reward for future *T* steps, $E[\sum_{t=0}^{T}\gamma^{t}r_{t}]$, where γ is a discount factor (0≤γ<1) for the infinite-horizon discounted model.

For effective agent learning, the estimation of a state-value function for a state *s* is critical; $V_{\pi }\left(s\right)=E_{\pi }\left[\sum_{k=0}^{\infty }\gamma^{t}R\left(s_{t+k+1}\right)|S_{t}=s\right]$ at a time step *t*. Additionally, suppose that a certain action, *a*, is taken in the state *s*; then, an action-value Q-function can be defined as $q_{\pi }\left(s,a\right)=E_{\pi }\left[\sum_{k=0}^{\infty }\gamma^{t}R^{a}\left(s_{t+k+1}\right)|S_{t}=s,A_{t}=a\right]$. According to the Bellman optimality equation, the optimal value function, $V_{*}\left(s\right)$, can be decomposed recursively as $V_{*}\left(s\right)=\max_{a}E\left[R_{t+1}+\gamma V_{*}\left(s_{t+1}\right)|S_{t}=s,A_{t}=a\right]$, which tells us that the expected return from the best action is the same as the state value of an optimal policy.

### 3.1. Deep Q-Learning

As the state and action spaces become larger and continuous, function approximation is mandatory for Q-learning instead of using a legacy tabular form of actions and Q-values. Although the combination of RL and neural networks was considered a long time ago, it is only very recently that DRL algorithms based on deep neural networks (DNNs) has received much attention instead of the linear function approximation [20,64]. DNNs represent a function with higher complexity by employing a deep hierarchical layer architecture that constitutes a non-linear information processing unit. Deep learning approximates such a mapping function for statistical curve fitting with labeled training datasets.

The DRL utilizes the training process of the DNN based on datasets which can improve learning speed and performance without the MDP model information (the *R* and $P_{ss^{\prime }}$ are unknown). The DRL induces a policy based on a value function, $V_{\pi }\left(s\right)$, approximated by the DNN, which is trained using the batch of samples $\left(S,A,R,S^{\prime }\right)$ that an agent collects by interacting with the environment. In a sequence of discrete time, {t=0,1,2,…}, the agent selects an ε-greedy action for the maximum reward given by $V_{\pi }\left(s\right)$; the ε provides randomness to explore and avoid the local minimum.

Mnih et al. introduced the deep Q-network (DQN) in [22], which is a seminal work for Q-function approximation based on DNNs. In particular, they addressed and solved two challenges in the DRL; first, the deep learning assumes that the data samples are iid (independent identically distributed), but actually the next state, $s^{\prime }$, is correlated with the current state, *s*, in the MDP. Second, the target model for training is non-stationary, as the model parameters θ are updated at every iteration. For this, the DQN adopts an experience-replay buffer for the training and separation of the main and target networks. The DQN updates θ of the main network by minimizing temporal-difference errors, $L\left(\theta \right)=Y_{t}-Q\left(s_{t},a_{t};\theta \right)$, where $Y_{t}=r_{t}+\gamma \cdot max_{a^{\prime }}\hat{Q}\left(s_{t+1},a^{\prime };\theta^{-}\right)$ and the state-action value function, Q(s,a;θ) are given by the target and main network, respectively. The target network is periodically updated by the main network.

### 3.2. Policy Gradient and Actor-Critic

The DQN is limited to high dimensional and continuous action spaces that demand iterative optimization processes at every step. Additionally, discretizing the continuous action values cannot avoid the curse of dimensionality due to a large number of actions, or, probably, loses important information of the action space from quantization.

Therefore, the policy gradient (PG) algorithm is used mostly for high dimensional and continuous actions [65,66], which adjusts the model parameter, θ, of a policy function in the direction of the stochastic policy gradient (SPG), $\nabla_{\theta }J\left(\pi_{\theta }\right)$.

$$\nabla_{\theta }J\left(\pi_{\theta }\right)=\int_{S}\rho^{\pi }\left(s\right)\int_{A}\nabla_{\theta }\pi_{\theta }\left(a|s\right)Q^{\pi }\left(s,a\right)\;dads$$

(1) $$=E_{s∼\rho^{\pi },a\;∼\pi_{\theta }}\left[\nabla_{\theta }\pi_{\theta }\left(a|s\right)Q^{\pi }\left(s,a\right)\right]$$

The PG algorithm [21] can be implemented by the actor-critic architecture, in which the actor stochastically updates the θ of the policy function while the critic evaluates the policy and updates the action-value function approximator, $Q^{w}\left(s,a\right)$, in such a direction as to minimize error, $\epsilon^{2}\left(w\right)=E_{s∼\rho^{\pi },a\;∼\pi_{\theta }}\left[{\left(Q^{w}\left(s,a\right)-Q^{\pi }\left(s,a\right)\right)}^{2}\right]$. As the dimension of action spaces increases, deterministic policy gradient (DPG) as a special case of the SPG is efficient to derive only the mean of the state spaces compared to the SPG, $\lim_{\sigma ↓0}\nabla_{\theta }J\left(\pi_{\mu_{\theta },\sigma }\right)=\nabla_{\theta }J\left(\mu_{\theta }\right)$.

## 4. System Model

In this section, we establish a mathematical system model of the HetNet with a mmWave backhaul mesh among SeNBs and MeNBs in which energy consumption and user traffic for the mmWave backhaul links and access links are formulated. In this model, we present dual objectives to minimize the energy while maximizing the user throughput. The symbols used in this model are described in Table 2.

### 4.1. Energy Consumption Model

The energy consumption of eNB *i* is composed of two folds: energy consumption from access links toward UEs and backhaul links toward other eNBs,

(2) $$\begin{array}{c} e_{i}=e_{i}^{AN}+e_{i}^{BN}, \end{array}$$

where energy consumption in the access network (AN) and backhaul network (BN) are $e_{i}^{AN}$ and $e_{i}^{BN}$, respectively.

#### 4.1.1. AN Energy Consumption

According to the linear approximation [67] between relative RF output power and the power consumption of an eNB, energy consumption for the access links can be derived as

(3) $$\begin{array}{c} \begin{array}{cc} e_{i}^{AN}= & P_{0_{i}}^{AN}+\Delta_{p}\cdot P_{out_{i}}^{AN}\;\forall i\in N \end{array} \end{array}$$

where $\Delta_{p}$ is a multiplier for load-dependent power consumption, which is different from the type of antenna (refer to Table 3) [67].

(4) $$\begin{array}{ccc} P_{out_{i}}^{AN} & = & P_{max_{i}}^{AN}\cdot F_{i}^{AN}=P_{max_{i}}^{AN}\cdot \\ & & \frac{1}{N_{RB_{i}}}\sum_{u\in U}\left\lceil \frac{f_{iu}^{u}x_{iu}^{u}}{N_{a_{iu}}^{AN}\cdot B_{RB}\cdot log_{2}\left(1+SINR_{iu}\right)}\right\rceil \end{array}$$

where the SINR is the signal-to-noise and interference ratio, $P_{out_{i}}^{AN}$ is the power consumption of the transceiver for the access links for all associated UEs, and  $0<P_{out_{i}}^{AN}\leq P_{max_{i}}^{AN}$. $P_{max_{i}}^{AN}$ is the maximum transmission power for the AN transceiver at the eNB *i*. The $P_{out_{i}}^{AN}$ can be scaled by the aggregated flow rate $F_{i}^{AN}$ against the link capacity, which is the same as the ratio of radio resource blocks (RB) used by all associated UEs to the total available RBs ($N_{RB_{i}}$); the number of used RBs can be calculated by dividing the sum of user data rate by the rate of a single RB (bandwidth $B_{RB}$ Hz). $N_{a_{iu}}^{AN}$ is the number of antenna for MIMO and $f_{iu}^{u}$ is the data rate for each UE. $x_{iu}^{u}$ is an integer value {0,1} to indicate the UE association with the eNB *i*.

As shown in Equation (3), the eNB has a statically minimum non-zero output power of the transceiver, $P_{0_{i}}^{AN}$, although there is no associated UE. Accordingly, switching off unused eNBs is critical to save energy. Table 3 shows experimental values for the aforementioned parameters in this study, such as $P_{max_{i}}^{AN}$ and $P_{0_{i}}^{AN}$.

#### 4.1.2. BN Energy Consumption

The energy consumption of a BH link can be formulated similarly to the AN link: (i) static power ($P_{0_{i}}^{BH}$) of a transceiver for each backhaul link toward a next-hop eNB *j*, and (ii) dynamic power by the amount of aggregated user data rate that travels over that link:

(5) $$\begin{array}{c} e_{ij}^{BH}=P_{0_{i}}^{BH}+P_{out_{i}}^{BH_{j}} \end{array}$$

where $P_{0_{i}}^{BH}$ represents the minimum non-zero static power of each BH transceiver at eNB *i*.

The dynamic power $P_{out_{i}}^{BH}$ of a mmWave backhaul link is derived by the multiplication of the band-wide transmission power $P_{t_{i}}^{BH_{j}}$ and bandwidth efficiency, as below:

(6) $$P_{out_{i}}^{BH_{j}}=P_{t_{i}}^{BH_{j}}\cdot F_{i}^{BH_{j}}=P_{t_{i}}^{BH_{j}}\cdot \frac{\sum_{u\in U}f_{ij}^{u}x_{ij}^{u}}{B_{ij}^{max}}$$

where $B_{ij}^{max}$ is the maximum data rate for a backhaul link ij. The integer value $x_{ij}^{u}$ indicates routing information if a data flow of a user *u* uses the backhaul link ij or not.

(7) $$\begin{array}{ccc} P_{t_{i}}^{BH_{j}} & = & SNR+N_{th}+NF+PL+L_{t}\\ & & +L_{r}-G_{t}-G_{r}+L_{m}, \end{array}$$

where SNR is the signal-to-noise ratio satisfying $B_{ij}^{max}$, $N_{th}$ stands for the thermal noise, NF stands for the noise figure and PL represents the free-space path loss. The parameters $L_{t}$ and $L_{r}$ represent the transmitter and receiver losses, respectively, while $G_{t}$ and $G_{r}$ are the transmitter/receiver antenna gains and $L_{m}$ is the link margin.

The maximum transmitted power of a transceiver operating at frequency $f_{BH}$ may be given by

(8) $${P_{max_{i,BH}}}_{\left(dBm\right)}={{EIRP}_{max}}_{\left(dBm\right)}+{T_{x_{loss}}}_{\left(dB\right)}-{G_{Tx}}_{\left(dBi\right)},$$

where ${EIRP}_{max}$ denotes the maximum equivalent isotropically radiated power, and $P_{max_{i}}^{BH}$ is configured as 224 mW according to specifications in [70], as shown in Table 3.

Total energy consumption of the BN is the sum of the energy consumption of the available backhaul links, as below.

(9) $$\begin{array}{c} e_{i}^{BN}=\sum_{j\in N}e_{ij}^{BH} \end{array}$$

As a consequence, the energy consumption of each eNB depends on user data flows and the static power consumption. Control message unicast or broadcast in the cell can consume extra energy in addition to the user traffic. In this study, we ignore energy consumption from the control overhead that is relatively less than the bearer. In the following section, therefore, we define several constraints to switch on or off the SeNBs based on the presence of the data flows.

### 4.2. Switch On and Off Model

We introduce two binary variables, $s_{i}^{AN}$ and $s_{i}^{BN}$, that indicate whether the AN link and the BH link, respectively, is powered on or off at node *i*; that is:

(10) $$s_{i}^{AN}=\left\{\begin{array}{c} 1\;\mathrm{when}\;\mathrm{AN}\;\mathrm{at}\;i\;\mathrm{is}\;\mathrm{powered}\;\mathrm{on},\;\forall i\in N\\ 0\;\mathrm{when}\;\mathrm{AN}\;\mathrm{at}\;i\;\mathrm{is}\;\mathrm{powered}\;\mathrm{off},\;\forall i\in N \end{array}\right.$$

(11) $$s_{i}^{BN}=\left\{\begin{array}{c} 1\;\mathrm{when}\;\mathrm{all}\;\mathrm{BH}\;\mathrm{at}\;i\;\mathrm{are}\;\mathrm{powered}\;\mathrm{on},\;\forall i\in N\\ 0\;\mathrm{when}\;\mathrm{all}\;\mathrm{BH}\;\mathrm{at}\;i\;\mathrm{are}\;\mathrm{powered}\;\mathrm{off},\;\forall i\in N \end{array}\right.$$

The power status of the AN and BN, $s_{i}^{AN}$ and $s_{i}^{BN}$, is decided by the use of access or backhaul links. Accordingly, switch variables for AN and BN are configured by the presence of data flows, as below:

(12) $$s_{i}^{AN}\leq \sum_{u\in U}f_{ij}^{u}\;\forall i\in S,\;\forall \left(i,j\right)\in {L_{\mathrm{AN}}}^{i}$$

(13) $$s_{i}^{BN}\leq \sum_{u\in U}f_{ij}^{u}\;\forall i\in S,\;\forall \left(i,j\right)\in {L_{\mathrm{BH}}}^{i}$$

For the multi-hop routing path of the user flows, a link (i,j) of power-off eNB *i* cannot be used as $x_{ij}^{u}=0$:

(14) $$\begin{array}{cc} \; & \sum_{u\in U}x_{ij}^{u}\leq s_{i}^{AN}\cdot \varsigma ,\;\forall \left(i,j\right)\in {L_{\mathrm{AN}}}^{i}\\ \; & \sum_{u\in U}x_{ij}^{u}\leq s_{i}^{BN}\cdot \varsigma ,\;\forall \left(i,j\right)\in {L_{\mathrm{BH}}}^{i} \end{array}$$

where ς is a big number (i.e., 10${}^{8}$).

### 4.3. Multi-Hop Routing Model

In this section, routing constraints are given for user data flows in the mmWave backhaul mesh network. First, a user data flow should satisfy the flow conservation rule in Equation (15). Second, a user data flow travels along a single path rather than multiple paths in Equation (16); in this study, we only consider single connectivity rather than dual connectivity. Third, an UE therefore has to associate with only one eNB in Equation (17).

(15) $$\begin{array}{c} \sum_{j\in N}f_{ij}^{u}-\sum_{j\in N}f_{ji}^{u}=\left\{\begin{array}{cc} R^{u},\; & \;\mathrm{if}\;i=\;\mathrm{source}\;\\ -R^{u},\; & \;\mathrm{if}\;i=\;\mathrm{sink}\;\\ 0 & \;\mathrm{otherwise}\; \end{array}\right.\\ \forall u\in U,\forall i\in N, \end{array}$$

where $R^{u}$ represents the demanded data rate of each UE *u*.

(16) $$\begin{array}{c} \sum_{j\in N}x_{ij}^{u}-\sum_{j\in N}x_{ji}^{u}=\left\{\begin{array}{cc} 1,\; & \;\mathrm{if}\;i=\;\mathrm{source}\;\\ -1,\; & \;\mathrm{if}\;i=\;\mathrm{sink}\;\\ 0 & \;\mathrm{otherwise}\; \end{array}\right.\\ \forall u\in U,\forall i\in N, \end{array}$$

where $x_{ij}^{u}=\left\{0,1\right\}$ indicates the routing information of a user data flow, $f^{u}$.

(17) $$\sum_{\left(iu\right)\in L_{\mathrm{AN}}}x_{iu}^{u}=1,\;\forall u\in U$$

### 4.4. Link Capacity and Scheduling Model

For capacity constraint, the data rate of each user flow and aggregated flows must be less than the access and backhaul link capacity. For instance, when more than one UE connects to the same eNB, they have to share the capacity on that access link.

Therefore, the AN capacity constraint is given as follows:

(18) $$\sum_{u\in U}\sum_{\left(i,u\right)\in L_{\mathrm{AN}}}f_{iu}^{u}\leq C_{i}^{max},\;\forall i\in N,$$

where $C_{i}^{max}$ is the maximum capacity of eNB *i* as the access link capacity.

Additionally, the sum of the user flows on a given BH link is limited by the maximum capacity of the BH link:

(19) $$\sum_{u\in U}f_{ij}^{u}\leq c_{ij},\;\forall \left(i,j\right)\in L_{\mathrm{BH}}$$

where $L_{\mathrm{BH}}$ represents a set of BH links.

In the mmWave backhaul mesh network, we have to schedule transmissions among all links in the set of interference links, (i,j)∈I. For duplex, first we adopt time division duplex (TDD), which is used to separate transmission and reception on a BH link (i.e., different time slots are assigned for the transmission from eNB *i* to *j* and for the transmission from eNB *j* to *i*). Similarly, time division multiplexing (TDM) is used to schedule transmissions among adjacent BH links. The following constraint ensures that the capacity of each BH link is shared among adjacent interferenced BH links:

(20) $$\sum_{u\in U}\left(\frac{f_{ij}^{u}x_{ij}}{b_{ij}}+\sum_{\left(kl)\in I((ij)\right)}\frac{f_{kl}^{u}x_{kl}}{b_{kl}}\right)\leq 1,\;\forall i\;\mathrm{and}\;j\in N$$

The flow rate on the link (i,j) can increase at the given link capacity as the interference is reduced by switching off SeNBs with the interfering BH links (i,j)∈I.

### 4.5. Dual Objective Function

In this study, we have dual objectives, which are minimizing the total energy consumption of the HetNets while maximizing the sum of data rate $R_{u}$ of each user *u* with the aforementioned constraints:

(21) $$\begin{array}{c} \min\;\omega_{1}\sum_{i\in N}e_{i}-\omega_{2}\sum_{u\in U}R^{u} \end{array}$$

s.t.Equations (2)–(20)

where $\left\{\omega_{1},\omega_{2}\right\}$ is a scaling vector that is used to impose weight for each objective; $\omega_{1}$ and $\omega_{2}$ are for energy consumption and throughput, respectively.

## 5. Deep Multi-Objective Reinforcement Learning in mmWave HetNet

In this section, we solve the optimization problem in Equation (21), which is not only non-convex, but contains dual objectives that are conflicting to each other. We introduce the PPO and PDOLS algorithms to effectively search for efficient solutions in the Pareto front of the dual objectives.

### 5.1. Proximal Policy Optimization

The TRPO is a stochastic policy-based optimization technique that can guarantee updates in the direction of increasing performance within a trust region. Schulman et al. [23] proposed a new policy optimization algorithm following the TRPO, called the PPO algorithm [24]. After then, several algorithms such as TD3 [71] and soft actor critic (SAC) [25] have been proposed, but the PPO is still a popular algorithm with some advantages of the TRPO. The PPO is easy to implement, using only first-order optimization, and is able to solve the data efficiency problem while achieving a similar performance as the complicated TRPO.

In the TRPO, updates are conducted by a policy that maximizes the objective function (“surrogate” objective) within a specific constraint as below,

(22) $$\max_{\theta }E_{t}\left[\frac{\pi_{\theta }\left(a_{t}|s_{t}\right)}{\pi_{\theta_{old}}\left(a_{t}|s_{t}\right)}A_{t}\right]$$

(23) $$\mathrm{subject}\;\mathrm{to}\;E_{t}\left[\mathrm{KL}[\pi_{\theta_{old}}\left(\cdot |s_{t}\right),\pi_{\theta }\left(\cdot |s_{t}\right)]\right]\leq \delta$$

By applying the Kullback–Leibler divergence (KL) constraint between the old policy $\pi_{\theta_{old}}\left(a_{t}|s_{t}\right)$ and the current policy $\pi_{\theta }\left(a_{t}|s_{t}\right)$ in Equation (23), the TRPO can provide monotonical improvement to the $\pi_{\theta }\left(a_{t}|s_{t}\right)$ at each iteration and prevent excessive updates by limiting the range δ. However, it demands intensive computation for a rough solution that is infeasible to analyze. Instead, the constraint is relaxed by penalty with coefficient β in Equation (24), in which the surrogate objective forms a lower bound to guarantee the performance of the policy π.

(24) $$\max_{\theta }E_{t}\left[\frac{\pi_{\theta }\left(a_{t}|s_{t}\right)}{\pi_{\theta_{old}}\left(a_{t}|s_{t}\right)}A_{t}-\beta \;\mathrm{KL}\left[\pi_{\theta_{old}}\left(\cdot |s_{t}\right),\pi_{\theta }\left(\cdot |s_{t}\right)\right]\right]$$

However, it is difficult to choose a constant value of β that performs well across various problems. For this, a new surrogate object function of the PPO is proposed to emulate monotonous improvement of the TRPO. The new surrogate objective function is presented in Equation (25),

(25) $$L\left(\theta \right)=E_{t}\left[\min(r_{t}\left(\theta \right)A_{t},clip\left(r_{t}\left(\theta \right),1-\epsilon ,1+\epsilon \right)A_{t})\right]$$

Using the clip function, the PPO enables the surrogate objective function to avoid excessive policy updates while achieving similar performance to the TRPO. In addition, the PPO collects fixed-length *T* trajectory segments as a mini-batch and performs learning based on them repeatedly, which increases sample efficiency and learning stability.

For calculating $A_{t}$, a truncated version of generalized advantage estimation (GAE) is used,

(26) $$\hat{A_{t}}=\delta_{t}+\left(\gamma \lambda \right)\delta_{t+1}+\cdots +\cdots +{\left(\gamma \lambda \right)}^{T-t+1}\delta_{T-1},$$

(27) $$\;\delta_{t}=r_{t}+\gamma V(s_{t+1})-V\left(s_{t}\right).$$

Due to the high sample complexity (i.e., the number of training samples required for successfully learning) of our HetNet model that probably increases the number of necessary samples and their variance, we apply the truncated version of GAE, which provides stable and steady learning in the PPO algorithm [72]. GAE can enable monotonous increments in reward by reducing the sample variance through discount vector γ and λ like the TD(λ).

### 5.2. MDP of mmWave-Backhaul HetNets

In this section, we define a MDP model $\left(S,A,R^{a},P_{ss}^{a}\right)$ for our multi-objective optimization problem in the HetNets.

- State *S*: the state in the HetNet MDP is denoted by a traffic matrix that represents traffic load $v_{e}=\left[0,1\right]$ at access and backhaul links, which eventually determines throughput and energy consumption. In particular, we define a single representative state for all access links of a certain eNB instead of the individual state to reduce state information, since the AN energy consumption from transmission power, $P_{out_{i}}^{AN}$, is calculated by aggregated RBs of all associated users as shown in Equation (4). Accordingly, the vector size of the state space is $|L_{\mathrm{BH}}\left|+|N\right|$. We define the environment state, $s_{t}=\left\{v_{1},v_{2},\ldots ,v_{|L_{\mathrm{BH}}|},v_{|L_{\mathrm{BH}}|+1},\ldots ,v_{|L_{\mathrm{BH}}\left|+|N\right|}\right\}$, with $v_{e}$, as below:
  (28) $$\begin{array}{cccc} v_{e}= & \frac{1}{c_{ij}}\sum_{u\in U}f_{ij}^{u},\; & \left(i,j\right)\in L_{\mathrm{BH}}, & \;e=[1,|L_{\mathrm{BH}}|] \end{array}$$
  (29) $$\begin{array}{cccc} = & \frac{1}{C_{i}^{max}}\sum_{u\in U}f_{ij}^{u},\; & \left(i,j\right)\in L_{\mathrm{AN}}, & \;e=[|L_{\mathrm{BH}}|+1,|L_{\mathrm{BH}}|+|N|] \end{array}$$
  where the index *e* of each link (i,j) is given by the environment at the beginning of the learning phase;
- Action *A*: the agent action is routing and association of user flows, which actually decides a set of $x_{ij}$ binary variables, as discussed in Equations (16) and (17). However, such discrete action space grows exponentially by the number of the links, in which convergence of the learning algorithm is rarely guaranteed and large memory is required for computation. Instead, we consider a weight matrix ($a_{t}\in R^{\left|L\right|}$) of all links for all user flows, with which each flow finds a path using a link-state routing algorithm (e.g., the Dijikstra algorithm). Accordingly, the space complexity decreases from $O(2^{\left|L\right|})$ to O(|L|). All actions for the links can be defined as below:
  (30) $$a_{t}=\left\{w_{ij}|w_{ij}\in R,\;\left(i,j\right)\in L\right\}$$
  Unfortunately, such a shortest path algorithm leads most of users to select a MeNB’s AN link as a single-hop path; cumulative weights along a multi-hop path are mostly higher than for a single hop. This prevents the DRL algorithm from exploring actions of multi-hop routing that may offer reward gain by increasing user throughput, $\sum_{u\in U}R^{u}$, more than the cost of energy consumption, $\sum_{i\in N}e_{i}$.
  Therefore, we limit the number of user flows for the MeNB in the routing algorithm that admits the user flows to the MeNB only if the MeNB has available RBs, $\sum f_{ij}^{u}\leq C_{i}^{max},i\in M,j\in U$. Otherwise, users find multi-hop paths through SeNBs in the algorithm;
- Reward *R*: the reward is given by the objective function of Equation (21). Thus, we change the minimization objective to maximization by multiplying Equation (21) by −1. For normalization, the sum rate of all UE flows and corresponding eNB energy consumption are divided by the sum of the maximum data rate and maximum energy consumption. Subsequently, the reward can be written in Equation (31) as
  (31) $$r_{t}=-\omega_{1}\cdot r_{e}+\omega_{2}\cdot r_{d},$$
  where $r_{e}$ and $r_{d}$ represent $\sum_{i\in N}\frac{e_{i}}{e_{max}}$ and $\frac{1}{\left|N\right|}\cdot \sum_{u\in U}\frac{R^{u}}{d_{u}}$, respectively.

### 5.3. PPO-Based DRL for HetNet Optimization

The aforementioned MDP model of our HetNet optimization has continuous state and action spaces; thus, the PPO can effectively perform the exploration of solutions without the excessive updates in Equation (25). We implement the PPO-based DRL algorithm in Algorithm 1, which is based on the actor-critic architecture.
**Algorithm 1** Proposed PPO Solution for mmWave HetNet**Input:**  $\pi_{\theta },V_{\phi },\left\{\omega_{1},\omega_{2}\right\},Env$**Instruction:**  1:**for** iteration=1,2, ….,**do**2:    **for** iteration=1,2, …., *T* **do**3:        **for** iteration=1,2, …., $|L_{BH}\left|+|N\right|$ **do**4:           $s_{t}=s_{t}\cup v_{e}$5:        **end for**6:        $a_{t}=\pi_{\theta_{old}}\left(s_{t}\right)$7:        $\left[r_{e}^{t},r_{d}^{t}\right],s_{t+1}=Env\left(a_{t}\right)$8:        $r_{t}=-\omega_{1}\cdot r_{e}+\omega_{2}\cdot r_{d}$9:        $M=M\cup \{s_{t},a_{t},r_{t},s_{t+1}\}$10:        ${\hat{A}}_{t}=$ compute advantage estimate from Equation (26)11:    **end for**12:    **for** iteration=1,2, …., *K* **do**13:        update $\pi_{\theta }$ using Equation (32)14:        update $V_{\phi }$ using Equation (33)15:    **end for**16:    $\theta_{old}=\theta ,\phi_{old}=\phi$17:    Drop *M*18:**end for**

In the input of Algorithm 1, the actor network $\pi_{\theta }$ parametrized by θ provides a policy ($a_{t}$) according to the environmental state ($s_{t}$). Meanwhile the critic network presents the reward value ($V_{\phi }\left(s_{t}\right)$), which is parametrized by ϕ. At the beginning, the PPO collects total *T* trajectory tuples ($S,A,R,S^{\prime }$) (line 2–11), and subsequently, $\pi_{\theta }$ and $V_{\phi }$ are trained multiple K times with the *T* collected tuples (line 12–15). The parameters of $\pi_{\theta }$ and $V_{\phi }\left(s_{t}\right)$ are updated by Equations (32) and (33).

(32) $$\theta =\arg\max_{\theta }E_{t}\left[L\left(A_{t},\theta_{old}\right)\right]$$

where $L(A_{t},\theta_{old})$ is derived by Equation (25) at the given old parameter $\theta_{old}$.

(33) $$\phi ={\mathrm{Smooth}}_{L_{1}}\left(\right|V_{\phi }\left(s_{t}\right)-{\hat{V_{t}}}^{GAE(\gamma ,\lambda )}\left|\right)$$

where the ${\hat{V_{t}}}^{GAE(\gamma ,\lambda )}$ is a target value derived by Equation (26); that is, ${\hat{V_{t}}}^{GAE(\gamma ,\lambda )}=V_{\phi_{old}}\left(s_{t}\right)+\hat{A_{t}}$.

Since we implement both an actor network and a critic network, $\pi_{\theta }$ and $V_{\phi }\left(s_{t}\right)$ using multi-layer perceptrons(MLP), in the gradient update process, backward propagation is conducted; in this paper, we adopt ${\mathrm{Smooth}}_{L1}$ as an optimizer among Adagrad, Adam, ${\mathrm{Smooth}}_{L1}$, etc. Although the surrogate objective function of the PPO in Equation (25) is applied only to $\pi_{\theta }$, $V_{\phi }\left(s_{t}\right)$ is affected interactively within the actor-critic loop.Thereby, both policy and value can avoid excessive updates. The update process of the algorithm continues until the reward increases and converges to a certain level.

### 5.4. Multi-Objective Deep Reinforcement Learning

The PPO-based DRL algorithm can suffer from finding Pareto fronts in the multi-objective MDP (MOMDP) problem since it just learns a policy with a scalarized single objective which is unclear to evaluate each contribution of different objectives. As the reward of the MOMDP is a vector of *n* rewards of multi-objectives, $R\left(s_{t},a_{t}\right)=r_{t}\in R^{n}$[47], for the reward scalarization, simple linearization such as $F\left(V^{\pi },\omega \right)=\omega \cdot V^{\pi }$ can be used (i.e., convex combination of the policy values, $V^{\pi }$), where $V^{\pi }$ is a value vector for a policy, π, and ω is a weight vector for the importance of the objectives [48].

**Figure 2:**
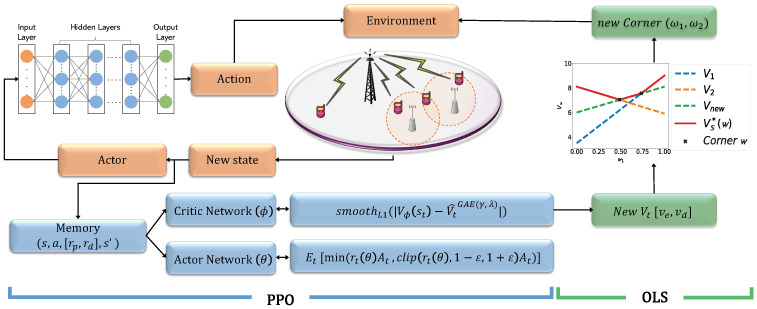
System architecture of the PPO-based deep OLS learning in mmWave HetNet.

Therefore, we propose the PDOLS algorithm to find an optimal solution for the MOMDP problem. Figure 2 depicts how the PPO and the OLS cooperate for the multi-objective HetNet problem. The OLS part provides a framework of the outer loop to handle possible weight vectors, while the PPO part provides actor-critic networks to update the policy and value. The outer loop incrementally constructs the *convex coverage set* (CCS) that is an intermediate approximated coverage set, S, by solving a series of single-objective MDPs scalarized by possible weight vectors, which eventually contains at least one optimal policy.

To reduce training efforts for all cases of weight vectors, the OLS manages corner weights that indicate break points in the piecewise linear CCS as a lower bound in addition to the S. Thus, the OLS selects the weight vector for training only among the corner weights. When a new corner weight, $\omega^{\prime }$, is discovered from the PPO learning, that is, $\exists v,F(V^{\pi },\omega^{\prime })>v$, $v\in V_{S}\left(\omega \right)=\left\{w\cdot V^{\pi }|V^{\pi }\in S\right\}$, all scalarized values below $F(V^{\pi },\omega^{\prime })$ are removed from S. Afterwards, the OLS selects the next corner weight in a priority queue for learning, as shown in Figure 2. The detailed procedures of the PDOLS are described in Algorithm 2.

The discovered corner weight $\omega_{1}$ and $\omega_{2}$ of energy consumption and throughput is used back for the PPO-based DRL to find a new lower bound of $V^{\pi }$ and its π in the Algorithm 2 (line 5–18). At that time, the reward value $r_{e}$ and $r_{d}$ of energy consumption and throughput can affect the creation of a set of $V_{S}^{*}\left(\omega \right)$. For instance, a new corner weight to be used for further learning and finding a new $V^{\pi }$ is rarely found if the reward gap between two objectives is large. Therefore, we scale down the reward value instead of the original value from environment in order to increase the probability of finding the new corner weights (line 10). ${\hat{A}}_{t}$ is calculated through Equation (26) and ${\hat{A}}_{t}\in A^{2}$ as $r_{t}\in R^{2}$ (line 12–13). To reflect the corner weight from the OLS in ${\hat{A}}_{t}$, ${\hat{A}}_{t}$ is updated by multiplying [$A_{e}^{t}$, $A_{d}^{t}$] and [$\omega_{1}$, $\omega_{2}$] (line 13). When the convergence is achieved in the PPO learning process, the PPO sends a new $V_{t}\left[v_{e},v_{d}\right]$ to the OLS (line 18).
**Algorithm 2** PPO-Based Deep Optimistic Linear Support1:Initialization:2:S = partial CSS, which is composed of $V_{t}$ obtained after the PPO learning.3:W = corner weights, which is obtained from S.4:Q = priority queue of weights for the multi-objective, where the weights form a tuple along with their importance (i.e., ($[\omega_{1}^{t},\omega_{2}^{t}],I$)).**Instruction:**  5:$\omega_{t}=Q$.pop()6:**for** iteration=1,2, ….,**do**7:    **for** iteration=1,2, …., *T* **do**8:        $a_{t}=\pi_{\theta_{old}}\left(s_{t}\right)$9:        $\left[r_{e}^{t},r_{d}^{t}\right],s_{t+1}=Env\left(a_{t}\right)$10:        Reduce scaling of $\left[r_{e}^{t},r_{d}^{t}\right]$11:        $M=M\cup \{s_{t},a_{t},\left[r_{e}^{t},r_{d}^{t}\right],s_{t+1}\}$12:        $[A_{e}^{t},A_{d}^{t}]=$ compute advantage estimate from Equation (26)13:        $\hat{A_{t}}=\hat{A_{t}}\cup \left\{\left[A_{e}^{t},A_{d}^{t}\right]\times \left[\omega_{1}^{t},\omega_{2}^{t}\right]\right\}$14:    **end for**15:    Optimize surrogate *L* and wrt θ from $\hat{A_{t}}$, with *K* epochs   16:    Optimize $V_{\phi }$ and wrt ϕ from ${\hat{V_{t}}}^{GAE(\gamma ,\lambda )}$, with *K* epochs17:$\;\;\;\theta_{old}=\theta ,\phi_{old}=\phi$18:**end for** when convergence19:$V_{t}=V_{\phi }\left(s\right)$20:$W=W\cup \omega_{t}$21:**if**$\;\omega_{t}\cdot V_{t}>\sum_{U\in S}\omega_{t}\cdot U$ **then**22:    S = remove obsolete $V_{del}$ due to new $V_{t}$23:    $\omega_{c}$ = new corner weight from S24:    $S=S\cup V_{t}$25:    Q = remove obsolete $\omega_{del}$ due to new $\omega_{c}$26:    **for** iteration=1,2, …., $\omega_{c}$ **do**27:        **if** estimate improvement of $(\omega^{\prime },W,S)>\tau$ **then**28:           $Q=Q\cup \omega^{\prime }$29:        **end if**30:    **end for**31:**end if**32:**if**Q is not empty **then**33:    go back to line 134:**end if**

The priority queue of the weights, Q, is initially configured with extreme weights (i.e., [0,1],[1,0]) and updated whenever a new corner weight is found. The priority is determined according to the distance between $F(V^{\pi },\omega^{\prime })$ of the new corner weight $\omega^{\prime }$ and a line made by values of two adjacent corner weights on both sides of the new corner weight. In other words, the priority is proportional to the degree of convexity downward in $V_{S}^{*}\left(\omega \right)$.

The OLS removes obsolete $V_{del}$ and $\omega_{del}$ when creating a new $V_{S}^{*}\left(\omega \right)$ (line 22, 25). Depending on the improvement of the new corner weight, the OLS decides whether to add it to the Q by comparing to a threshold τ (line 26–28). We set the τ to 0 to train aggressively for all discovered corner weights to find optimal values. Finally, the PPO and OLS stop processing if no new corner weight is found and Q is empty (line 32–33).

## 6. Experiment

**Figure 3:**
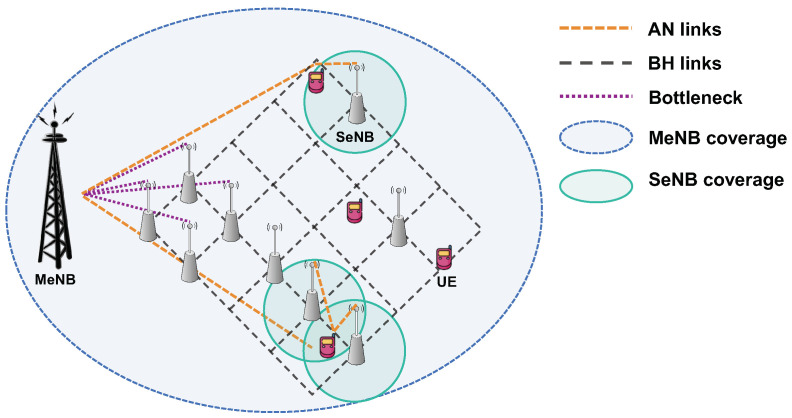
Experimental HetNet topology.

In this section, we evaluate the performance in terms of energy saving and user throughput, comparing algorithms proposed in the previous section. We establish an experimental environment with 1 MeNB and 25 SeNBs that form a backhaul mesh network as depicted in Figure 3, where the mmWave BH links (i.e., gray dashed lines in Figure 3) connect the SeNBs to each other or to the MeNB for Internet access. There are only 4 SeNBs reachable to the MeNB, which thus limits the sum rate of all data flows below the sum of their BH link capacity. Therefore, we assume that each UE, *u*, demands a maximum 14 Mbps data rate ($d_{u}$) in this experiment with the 100 UEs and last mile 4 SeNBs since those bottleneck BH links (i.e., the purple dot line in Figure 3) allow 14 Mbps per UE. To support a greater UE data rate, we can increase the BH link bandwidth or place more SeNBs reachable to the MeNB gateway.

A total of 100 UEs are randomly dropped over the MeNB and SeNB coverage area, where the SeNBs are apart by 100 meters and their cell coverage is more than 80 meters. Accordingly, the UEs have more than one SeNB to associate with, in addition to the universal MeNB, depending on their location. Both the MeNB and SeNBs provide microwave link access, denoted by AN links in Figure 3. The access and BH link is configured as in Table 3 for our experiment. In our study, the training and model update are performed interactively with the network simulator environment based on parameters specified in 3GPP standard and related works [68,69].

We build actor-critic networks using a DNN with 2 hidden layers (64 × 64 perceptrons) of a fully-connected neural network to estimate the policy and value, respectively. The actor network for policy receives the input of the state field and returns the action field as output as defined in Section 5.2. On the other hand, the critic network for value is designed differently according to the PPO and PDOLS algorithm. Both algorithms receive the same input for the state field, but the PPO-based critic returns only one value, while the PDOLS-based critic returns two values of the dual objectives. Detailed parameters for the DRL are introduced in Table 4. For this experiment, we used the pyTorch library on a Linux 20.04 server equipped with Intel CPU i7-9700KF, GPU GeForce RTX 2080 and 32 GB RAM.

**Figure 4:**
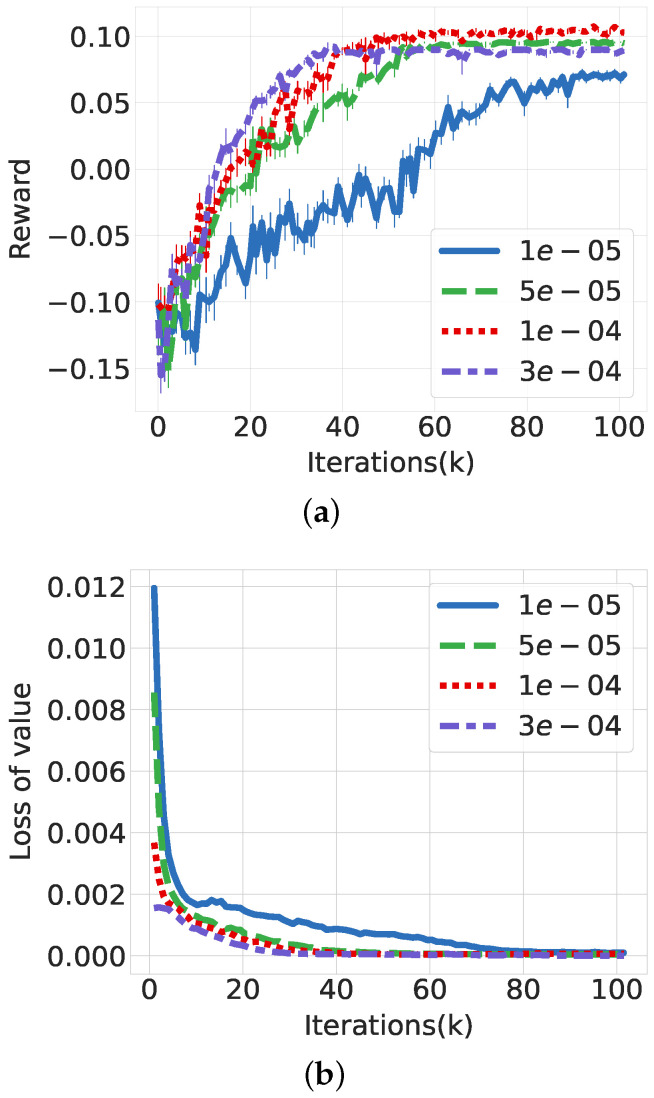

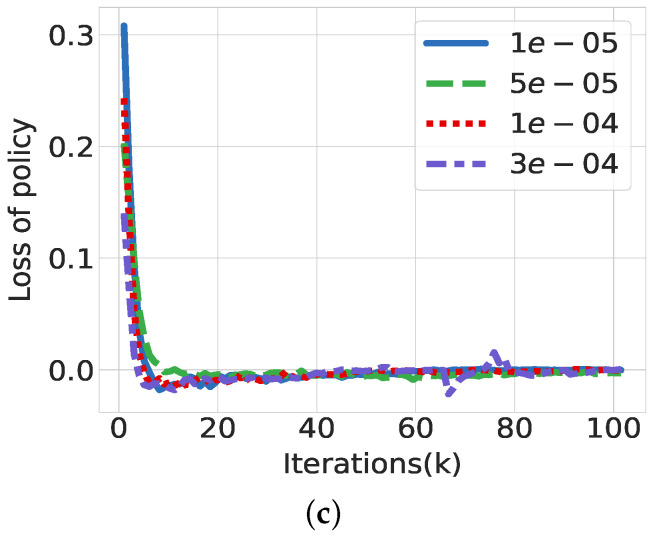
Performance evaluation of PPO according to learning rate. (**a**) Reward convergence. (**b**) Value loss. (**c**) Policy loss.

First, we evaluate the performance of the PPO-based DRL algorithm in the HetNet environment in terms of learning speed and convergence. For this, we configure the weight vector of energy consumption and data rate as $\omega_{1}=0.5$ and $\omega_{2}=0.5$, respectively, and the UE demand rate as 14 Mbps. Figure 4a shows the performance with varying learning rates from 1 × 10^−5^ to 3 × 10^−4^. The PPO algorithm shows good convergence of reward as training iterations continue, regardless of learning rate. The reward increases exponentially during the initial training iterations and becomes saturated after 40 K training iterations. The higher learning rate accelerates the reward convergence, but it skips over the better local minimum and is trapped in another; when the learning rate increases from 1 × $10^{-4}$ to 3 × $10^{-4}$, the converged reward decreases from 0.104 to 0.0899. The loss for the value and policy can be seen in Figure 4b,c, respectively. The loss of value and policy decreases drastically as the training iterations continue. Policy learning can avoid excessive learning owing to clipping of the PPO, which leads the policy loss to be comparable regardless of the learning rate. Additionally, the value loss follows the policy loss through the actor-critic interactions.

**Figure 5:**
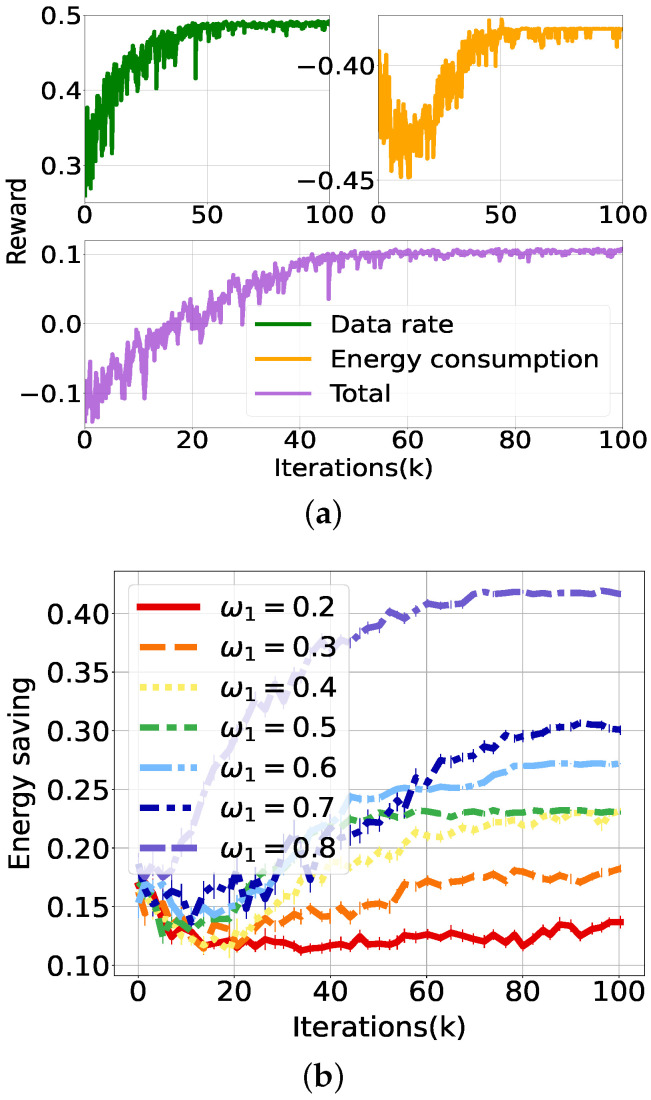

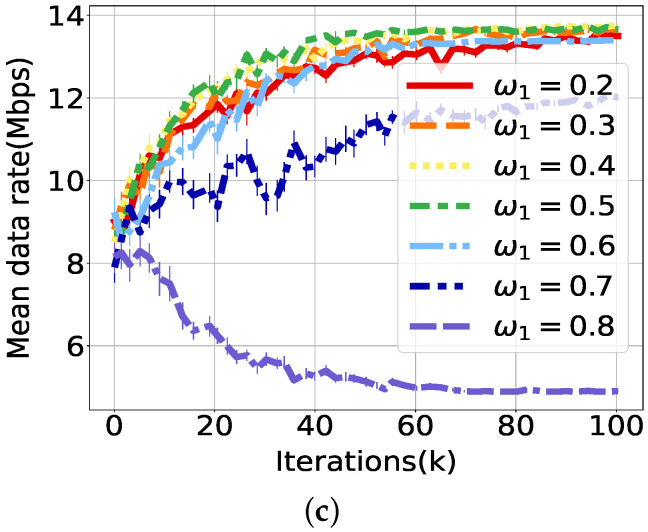
Performance evaluation of PPO according to reward weight ($\omega_{2}=1-\omega_{1}$). (**a**) Scalarized reward. (**b**) Energy savings. (**c**) Average data rate per UE.

Figure 5 shows evaluations on learning performance with varying reward weights ($\omega_{1}$, $\omega_{2}$). For this experiment, we configure the learning rate as 1 × $10^{-4}$, which shows the fastest convergence with the highest reward. In Figure 5a, rewards from energy consumption and user throughput converge at 50 K training iterations with reward weight ($\omega_{1}=0.5,\omega_{2}=0.5$).

Figure 5b,c show that the energy saving (i.e., 1-consumed energy/maximum energy) and mean data rate converge at different iterations according to the reward weight; the reward convergence is achieved at an average of 80 K training iterations, about 21.5 min on our server for each weight value. To find the optimal solutions, iterative learning for all possible weight vectors is needed. Therefore, the computation delay depends on the granularity of the weight values to explore; this experiment demands a total of 80 K · 7 iterations.

System performance varies with $\omega_{1}$ of the energy consumption from 0.2 to 0.8 and $\omega_{2}$ of the UE’s data rate, $1-\omega_{1}$. When $\omega_{1}$ is set to 0.8, the maximum energy saving is achieved by 0.419, while the UE’s data rate is only 4.89 Mbps as a minimum value, because of their trade-off relationship. Contrarily, the minimum energy saving, 0.134, allows the maximum data rate, 13.7 Mbps, with $\omega_{1}=0.2$. Consequently, the optimal weight for maximum reward is found to be $\omega_{1}=0.6$ and $\omega_{2}=0.4$, which results in an energy savings of 0.272 and a UE data rate of 13.39 Mbps.

**Figure 6:**
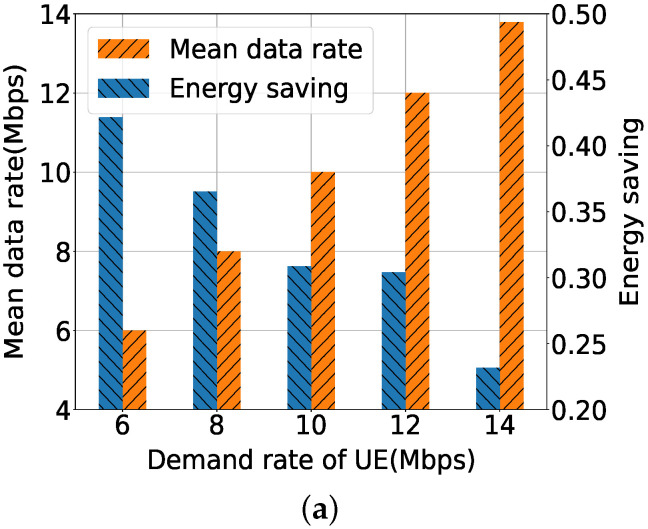

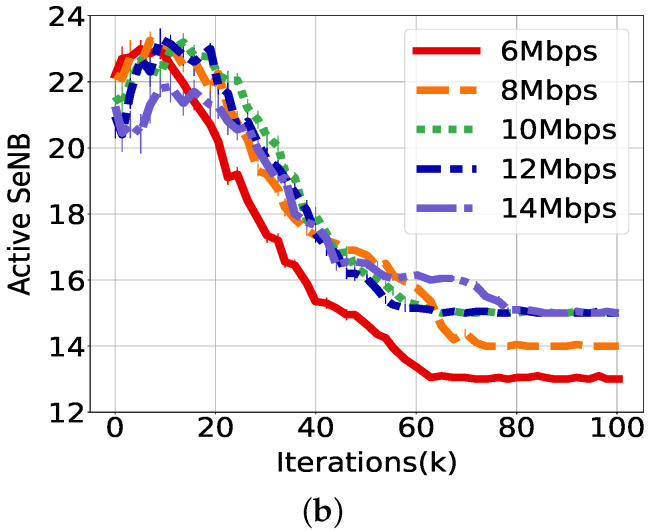
Performance evaluation of PDOLS according to UE’s demand rate. (**a**) Average data rate per UE. (**b**) Number of active SeNBs.

Next, we evaluate the PDOLS algorithm to find the optimal value and weight in a HetNet environment with a varying demand rate and number of UEs. In Figure 6a, the mean data rate satisfies most of all demand rates except for 14 Mbps: 6, 8, 10, 12, and 13.39 Mbps. The energy savings of the HetNet is inversely proportional to the demand rate: 0.42, 0.37, 0.31, 0.30, and 0.23. For these values, the $\omega_{1}$ of the optimal weight is 0.79, 0.72, 0.65, 0.64 and 0.57, with respect to each data rate.

Figure 6b shows the change of the active SeNBs during the learning procedure. Most of the 25 SeNBs are turned on at the beginning of learning, but after 80K iterations, almost 10–12 SeNBs are switched off according to the UE’s demand rate. For the higher demand rate, more SeNBs are active to support the user traffic. Although the number of active SeNBs is the same for 10, 12, and 14 Mbps, energy consumption increases, especially for the 14 Mbps in Figure 6a, as power consumption of the active links increases proportionally by user traffic.

**Figure 7:**
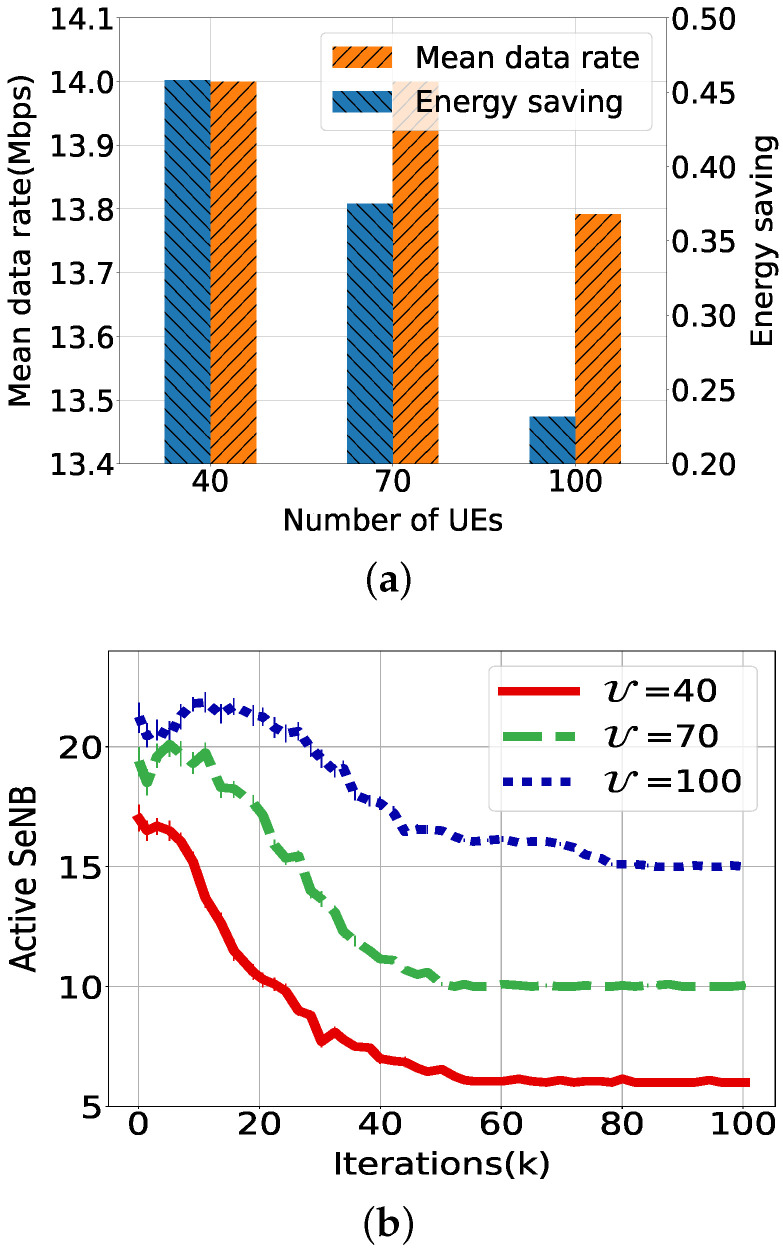
Performance evaluation of PDOLS according to the number of distributed UEs. (**a**) Average data rate per UE. (**b**) Number of active SeNBs.

We evaluate the performance of the PDOLS again with different numbers of UEs such as 40, 70 and 100, where the demand data rate is configured to be 14 Mbps. Figure 7a shows that both energy savings and the sum of the data rate increase as the number of UEs decreases. Accordingly, the user demand rate is mostly satisfied, except for 100 UEs. The energy saving is 0.46, 0.38, and 0.2, respectively, for each number of UEs. The corresponding active SeNBs are 6, 10, and 15, as shown in Figure 7b. Here, $\omega_{1}$ of the optimal weight is found to be 0.8, 0.66, and 0.57 for each case. For 40 UEs, the number of active SeNBs is around 18 initially and decreases to up to 6 SeNBs, as data flows of many UEs use the same multi-hop paths provided by the active SeNBs. Otherwise, isolated UEs that have no path through the SeNBs directly access to the MeNB. Comparing the result of 100 UEs with 6 Mbps, we can conjecture that a higher number of UEs induces network-wide deployment, which consumes more RBs of the MeNB and transmission power for a smaller number of serving UEs.

**Figure 8:**
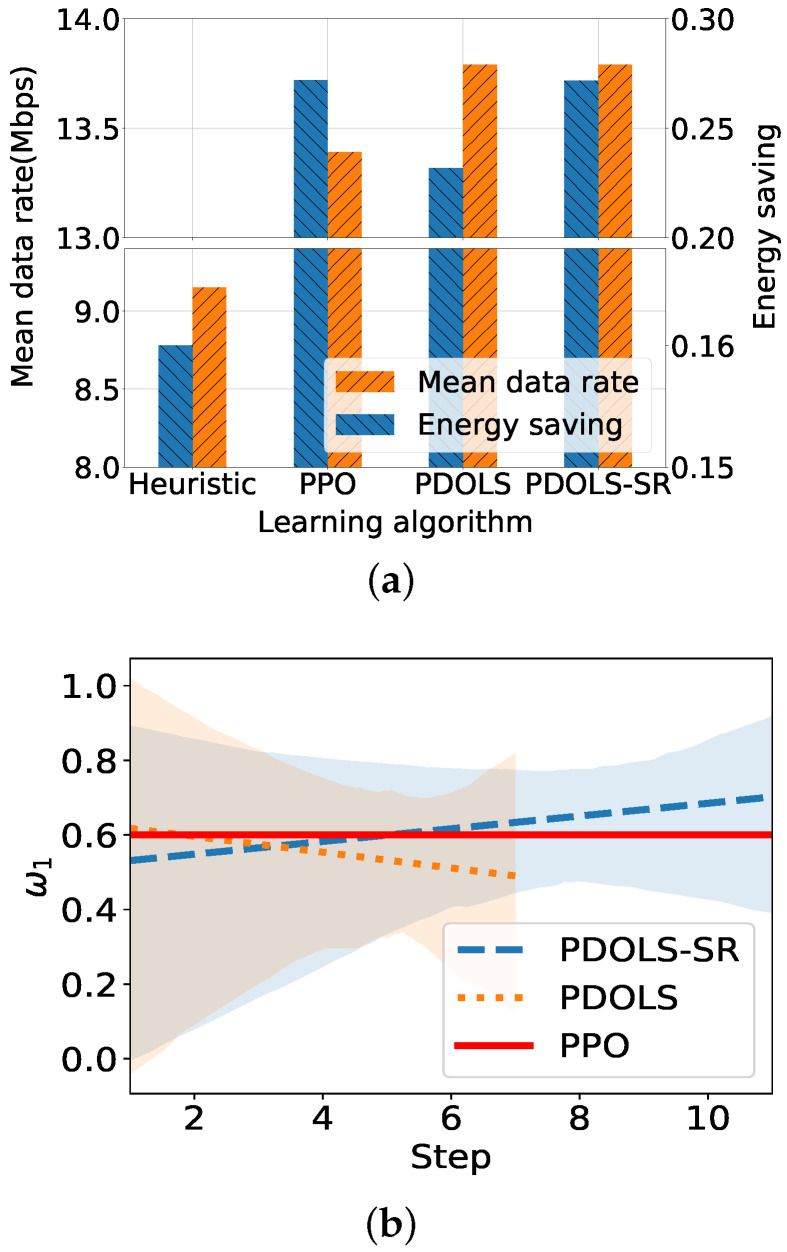
Performance comparison of proposed algorithms. (**a**) Average UE data rate and energy consumption. (**b**) Reward weight vector exploration.

Figure 8a compares the performance of the proposed algorithms discussed in Section 5, where the number of UEs and the demand rate are configured as 100 and 14 Mbps. A heuristic algorithm leads the UEs to associate with a less-loaded SeNB and use the shortest path to the MeNB gateway, which performs worse with energy savings of 0.16 and a data rate of 9.14 Mbps than others. Meanwhile, the PPO and PDOLS show comparable results of 0.27, 13.39 Mbps for the PPO and 0.23, 13.79 Mbps for the PDOLS, where the optimal weight for the PPO is selected manually after iterative executions with different weight vectors, while the PDOLS algorithm automatically searches for the optimal weight values. The PDOLS-SR outperforms other algorithms with 0.27 and 13.79 Mbps when the reward is scaled by 1/5.

Figure 8b shows the variation of corner weight in the OLS framework of the PDOLS. In our experiment, the PDOLS-SR conducts the training process 11 times (11 steps in the figure) to find the optimal weight, while the PDOLS does this only 7 times (7 steps). The PDOLS-SR can scavenge and explore more corner weights to find a near-optimal weight close to the PPO weight, 0.6 (the red solid line). The optimal $\omega_{1}$ of the PDOLS-SR is 0.5872, while the $\omega_{1}$ of the PDOLS is 0.5683. Further adjustment for downscaling of the reward, such as 1/10 or 1/15, only increases training time without notable performance enhancement.

## 7. Conclusions

In this paper, we solve a multi-objective optimization problem of throughput maximization and energy consumption minimization in a HetNet with a mmWave-backhaul mesh. For this, we implement a PPO-based DRL algorithm based on actor-critic architecture. However, the conventional PPO algorithm has limitations in its ability to cope with the multi-objective problem. Therefore, we propose PDOLS, which allows the PPO algorithm to interoperate with OLS as an outer loop to search for an optimal weight vector for the dual objectives. Experimental results show that the PPO-based DRL algorithm converges successfully with increasing rewards as training is iterated. Additionally, the learned solution of energy saving and user throughput is comparable to the CPLEX result. PDOLS can find a feasible weight vector for the dual objectives which is similar to the optimal weight that is identified manually using all possible combinations of the weight values.

## Figures and Tables

**Table 1:** DRL-empowered wireless communication and networking research.

References	Areas of DRL Studies on Wireless Communications
[26,27,28,29,30,31,32]	Cognitive radio and dynamic wireless channel selection increase spectral efficiency, which is typically a combinatoric problem of matching channels to nodes. Using DRL, agents can learn the optimal policy from the degree of interference as a reward for every action of channel selection.
[38,39,40,41]	The wireless link layer provides a media access scheme for multiple users which is realized in a MAC protocol. Several studies design the wireless MAC protocol based on the DRL algorithm, in which DRL agents learn an optimal transmission policy from the reward of contention resolution at a particular channel state.
[59,60,61]	A user association or handover algorithm for a serving base station affects throughput and QoS of each user. The DRL algorithm enables UEs to select an optimal base station based on past experience.
[33,34,35,36,37]	Wireless networks have various resources to be scheduled, such as radio block, channels, sequence codes, power, time slots, etc. Many of the scheduling problems have non-convex feasible set and user mobility, which makes the problems intractable. The DRL agents learn an optimal scheduling policy repeatedly from resource utilization against a chosen allocation.
[42,43,44,62,63]	Energy and power consumption is critical, especially for green wireless networking, mobile edge cloud networks and UAV networks. The DRL algorithm explores possible policies based on the reward of energy saving while guaranteeing throughput constraint.

**Table 2:** Parameters (P) and variables (V) used in the model.

Symbol	Description	
$B_{RB}$	Bandwidth for a RB	P
$c_{ij}$	Maximum capacity of link (*i*,*j*)	P
$C_{i}^{max}$	Maximum AN capacity of eNB *i*	P
$e_{i}$	Total energy consumption at node *i*	V
$f_{ij}^{u}$	Flow of UE *u* on link (*i*,*j*)	V
$x_{ij}^{u}$	Indicator if UE *u* uses link (*i*,*j*)	V
I	Set of interference links	P
L	Set of links	P
$L_{\mathrm{AN}}$	Set of AN links	P
$L_{\mathrm{BH}}$	Set of BH links	P
N	Set of eNB	P
M	Set of Macro eNB (MeNB)	P
S	Set of Small eNB (SeNB)	P
U	Set of UE	P
$N_{a_{iu}}$	Number of antennas (MIMO) for UE *u* at eNB *i*	P
$N_{RB_{i}}$	Number of RBs at node *i*	P
$P_{0_{i}}$	Static power at node *i*	P
$R^{u}$	User demand data rate *u*	V

**Table 3:** Parameters used for evaluation.

	MeNB-AN	SeNB-AN	BH Link
Frequency band (GHz)	2	2.6	60
Available BW (MHz)	20 ($BW_{PRB}$= 0.18 )	20 ($BW_{PRB}$= 0.18 )	1000 (10 × 100 MHz)
Antenna gain (dBi) ($G_{Tx}$, $G_{Rx}$)	<15	<15	36
$N_{ant_{i}}^{AN}$	4 (MIMO 4 × 4)	4 (MIMO 4 × 4)	1 for each active BH link
$P_{0_{i}}$ (W)	130	6.8	3.9
$P_{MAX}^{out}$ (W)	20	0.13	0.224
$\Delta_{p}$	4.7	4.0	not used
Distance-dep. Path Loss	$128.1+37.6\cdot \log_{10}\left(r\right)$ [68]	$140.7+36.7\cdot \log_{10}\left(r\right)$ [68]	Equations (6)–(11) in [69]

**Table 4:** Training hyperparameters.

Parameter	Value	Parameter	Value
γ	0.8	λ	0.8
Trajectory size	1024	Batch size	32
K epoch	10	Clipping range ϵ	0.2
Learning rate of actor	1 × $10^{-4}$	Learning rate of critic	1 × $10^{-4}$
Network initialization	HE	Optimization method	${\mathrm{Smooth}}_{L1}$

## Data Availability

The data used to support the findings of this study are available from the corresponding author upon request.
